# An Integrated Regulatory Network Reveals Pervasive Cross-Regulation among Transcription and Splicing Factors

**DOI:** 10.1371/journal.pcbi.1002603

**Published:** 2012-07-26

**Authors:** Idit Kosti, Predrag Radivojac, Yael Mandel-Gutfreund

**Affiliations:** 1 Faculty of Biology, Technion – Israel Institute of Technology, Haifa, Israel; 2 School of Informatics and Computing, Indiana University, Bloomington, Indiana, United States of America; MRC Laboratory of Molecular Biology, United Kingdom

## Abstract

Traditionally the gene expression pathway has been regarded as being comprised of independent steps, from RNA transcription to protein translation. To date there is increasing evidence of coupling between the different processes of the pathway, specifically between transcription and splicing. To study the interplay between these processes we derived a transcription-splicing integrated network. The nodes of the network included experimentally verified human proteins belonging to three groups of regulators: transcription factors, splicing factors and kinases. The nodes were wired by instances of predicted transcriptional and alternative splicing regulation. Analysis of the network indicated a pervasive cross-regulation among the nodes; specifically, splicing factors are significantly more connected by alternative splicing regulatory edges relative to the two other subgroups, while transcription factors are more extensively controlled by transcriptional regulation. Furthermore, we found that splicing factors are the most regulated of the three regulatory groups and are subject to extensive combinatorial control by alternative splicing and transcriptional regulation. Consistent with the network results, our bioinformatics analyses showed that the subgroup of kinases have the highest density of predicted phosphorylation sites. Overall, our systematic study reveals that an organizing principle in the logic of integrated networks favor the regulation of regulatory proteins by the specific regulation they conduct. Based on these results, we propose a new regulatory paradigm postulating that gene expression regulation of the master regulators in the cell is predominantly achieved by cross-regulation.

## Author Summary

The operation of a living cell depends on its ability to regulate its different functions. The master regulators in the cell are proteins, which control the function of many other genes by several mechanisms. Transcription factors can differentially activate or repress the transcription of genes by binding to their regulatory elements. A second major mechanism of gene expression regulation occurs at the level of alternative splicing. Alternative splicing is regulated by splicing factors that bind to short regulatory motifs on the RNA and dictate the final gene architecture. To date there is increasing evidence of coupling between transcription and splicing. In this study, we modeled a network integrating the two regulations. Analysis of the network indicated that splicing factors were more often regulated by alternative splicing while transcription factors were more extensively controlled by transcriptional regulation. Overall, we postulate that regulatory proteins in the cell are controlled preferentially by the specific regulation they conduct.

## Introduction

The operation of a functioning living cell depends on its ability to tightly regulate its different pathways. Most of this regulation is done by proteins that control the function of many other genes (or themselves in the case of autoregulation). Transcription factors (TFs) are the most abundant regulators in eukaryotic cells, controlling transcription of genes and playing a key role in many important cell functions [1]. Transcriptional regulation is usually a combinatorial effect of multiple TFs binding to regulatory elements in promoter or enhancer regions [2]. Splicing regulation is coordinated mainly by splicing factors (SFs) that bind to short regulatory motifs on the pre-mRNA, called splicing factor binding sites (SFBS), usually located in close proximity to the splice sites [3].

Over the past decade, there has been growing evidence of coupling and interconnectivity between the different steps of the gene expression pathway, specifically between RNA transcription and RNA processing [4]–[6]. The physical coupling between the different steps is known to be mediated by the CTD (C-Terminal Domain) of the largest subunit of RNA polymerase II that is recruited to the transcription complex by specific TFs [7]. This coupling is required both for efficient gene expression in higher eukaryotes and for enabling rapid response to diverse signaling events in the cell [8]. Alternative splicing (AS) events are known to play an important role in modulating the activity of TFs [9]. In a recent study, it was shown that an AS event within a TF mRNA encoding a DNA-binding protein alters the transcription regulatory network controlling the transition between pluripotency and differentiation in embryonic stem cells [10]. In another study, changes in AS patterns of TFs triggered by the activation of signal transduction pathways were shown to play an important role in development. In the latter study, the authors found that 40% of the genes that underwent AS changes also showed changes in transcription, supporting extensive cross-talk between the processes [11]. While the gene expression pathway is largely regulated by TFs and SFs, their activity is modulated by, among other things, post-translational modifications (PTMs). PTMs such as phosphorylation can switch the function of TFs, as was recently shown for CEBPB [12]. PTMs have also been shown to influence splice site selection, changing the spliceosome composition and changing the sub-cellular localization of regulatory proteins [13]. Since AS can remove or insert short fragments in a protein, it may also alter the phosphorylation pattern of the protein, thus suggesting another important role for AS in modulating the gene expression pathway.

Most recent knowledge from high-throughput experiments on transcriptional and splicing regulation provides a pair-wise relationship between a specific regulatory factor and its targets [14]–[16]. However, the complex interaction between the genes and the environment governing the cellular response cannot be understood at the level of individual interactions, but could rather emerge through the intricate interplay between the different regulators and their target genes. Understanding the complex interactions between the diverse regulators in the cell is crucial for unraveling the gene regulatory network in multicellular organisms, such as humans, as well as for helping to reveal the causes that render disease states. In recent years, many regulatory networks have been reconstructed to study this complex interplay between gene expression regulations. Most of the work in this direction has focused on transcription regulation in single-cell organisms, such as *E. coli* [17], [18] and *S. cerevisiae* [19]–[22]. In addition, several attempts have been made to integrate transcription networks into other regulatory networks. This approach has revealed elements of integration between a transcription regulatory network and splicing regulatory networks during the meiotic gene expression program in *S. cerevisiae* [23]. In a recent systematic study integrating transcription and phosphorylation networks in different species, the authors suggest a positive correlation between the species' complexity and the degree of cooperation in the network [24]. The complexity of the human regulatory network and a lack of experimental data explain why only a few studies to date have attempted to systematically explore regulatory networks in humans. One such study is the TF-microRNA network [25] based on predictions of transcription regulation and microRNA target regulation. This study revealed a scale-free behavior in which a small number of microRNA-TF pairs regulate large sets of common targets.

In this study, we focus on an integrated network of transcriptional and splicing regulation in humans. Our results show extensive wiring of the regulatory genes, specifically by AS regulation. Most strikingly, the network reveals that the subgroup of SFs has significantly higher density of splicing inedges (predicted alternative splicing regulatory interactions) compared to the subgroup of TFs, while transcriptional regulation is much more dense towards the TFs. Consistent with the network results, we found that the subgroup of kinases has significantly higher density of predicted phosphorylation sites relative to TFs and SFs. Taken together, our results indicate that cross-regulation within functional groups is significantly more prevalent than cross-talk regulation between groups, supporting the hypothesis that these functional groups are consistently under similar regulatory constraints. This new regulatory paradigm may point to a more general principle whereby a biological process is controlled predominantly by the entities that compose it.

## Results

### Combining splicing and transcriptional regulation in an integrated network

**Figure 1:**
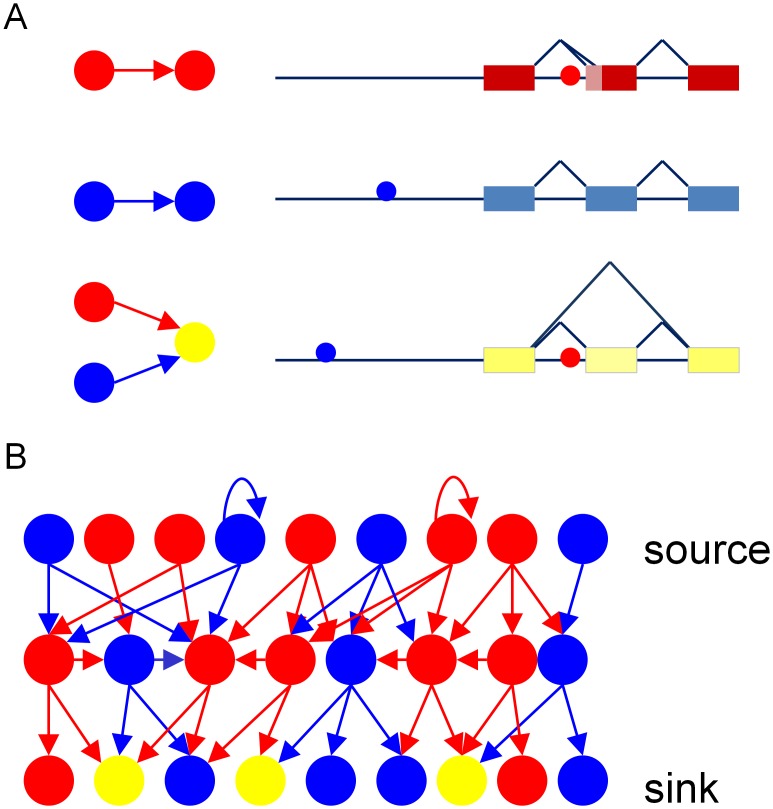
Schematic representation of the integrated network construction. (A) Three representative examples of different types of regulatory interactions in the integrated network. On the left are illustrations of the network interactions: red and blue arrows represent splicing and transcription regulation, respectively, accordingly large red and blue circles represent SF and TF nodes, respectively. On the right are sketches illustrating how each interaction was defined: small red and blue circles represent predicted splicing or transcription binding motifs, respectively. The color of the transcript illustrated is as follows: red for genes belonging to the SF group, blue for genes belonging to the TF group and yellow for genes belonging to the kinase group. The top row demonstrates the splicing regulatory interaction between two SFs, the middle row demonstrates the transcription regulatory interaction between two TFs, and the bottom row represents splicing and transcription interactions of a node belonging to the kinase group. (B) Illustration of the network architecture: the top row represents the source layer, including two types of nodes (SF, TF) with only outedges of splicing and transcription (source), the middle row includes two types of nodes (SF, TF) having both inedges and outedges (mixed layer), and the bottom row includes three types of nodes (SF, TF and kinases) having only inedges (sink).

To study the interplay between transcriptional and splicing regulation, we sought to concentrate on the main players in the process – the transcription and splicing factors. As a first step, we compiled a subset of experimentally verified transcription and splicing factors belonging to diverse protein families. In addition, we generated a non-redundant set of all human kinases [26]. Overall, the network was comprised of 257 nodes, of which 110 regulatory genes/proteins act as both regulators and targets in the network (20 SFs, and 90 TFs) and 147 nodes representing kinases acting as targets only. All of the nodes in the network were wired by two types of regulatory edges representing transcriptional and AS regulation. Full details regarding the network wiring is given in the Materials and Methods section. Briefly, an edge from a SF to any other factor was added if the gene coding to that factor had an AS event and a human-mouse conserved binding motif of the SF was found flanking the splicing event region (Figure 1A). To define a conserved SFBS, we employed our recently developed SFmap algorithm [27]. SFmap implements the COS(WR) algorithm, which computes the probability of a sequence to bind a given SFs based on the experimentally verified consensus motif, as well as information derived from its sequence environment and the overall conservation of the site. SFmap exploits two major attributes of functional SFBSs: their propensity to be grouped into clusters of similar motifs and their evolutionary conservation [28]. In our previous study, we showed that when employing SFmap on high-throughput experimental binding data obtained by cross-linking immunoprecipitation (CLIP) of two independent SF2/ASF (known also as SFRS1) [29] and NOVA [30] factors, we detected a significant enrichment of the predicted motifs in the experimentally selected sequences relative to a set of random sequences [28]. To verify the SFmap algorithm on more recent experimental data, we applied it on CLIP data for the polypyrimidine tract binding protein (PTB) [31] and binding data for the quaking (QKI) splicing factor obtained by the PAR-CLIP method [32]. Employing SFmap using the published motifs for the latter SFs, we predicted a significant hit of the motif in 75% and 71.4% of the binding targets of PTB and QKI, respectively. Here, again, we detected a significant enrichment of SFmap predicted binding sites among the experimentally selected sequences relative to random sequences (*p-value = 1e-16* for both PTB and QKI), reinforcing the strength of the method to detect true positive binding sites. Furthermore, in order to define an edge from a TF to any other factor, we followed the approach recently used for generating a microRNA-TF regulatory network [25]. We required the existence of a conserved binding motif of the regulating TF within the promoter region of the gene coding to the regulated factor based on the human/mouse/rat conserved sites extracted from UCSC TFBS sites table [33]. Overall, wiring the 257 nodes resulted in a complex three-layer network. The upper layer (‘source’) contained SFs and TFs with outedges regulating the middle and lower levels. The second, middle layer had a mixture of inedges and outedges of transcriptional and AS regulation to and from the factors. The third, lower layer (‘sink’) included TFs, SFs and kinases with transcription and splicing inedges (Figure 1B).

Next, we studied the network characteristics, concentrating on global properties, specifically clustering coefficient and sparseness. The clustering coefficient was 0.37, which is significantly higher compared to 1,000 random networks having a similar degree distribution (z-score = 41.51, *p-value*<2.2e-16). This suggests that the integrated splicing-transcription network tends to create tightly knit groups as was found for other regulatory networks [34], [35]. Furthermore, we calculated the sparseness of the network, which ranged from 0 to 1, 0 being the most sparse. In our integrated network, the sparseness was 0.1, consistent with the highly sparse nature of regulatory networks. This is presumably an adaptive feature that is more robust to loss of an edge in the course of evolution [35].

**Figure 2:**
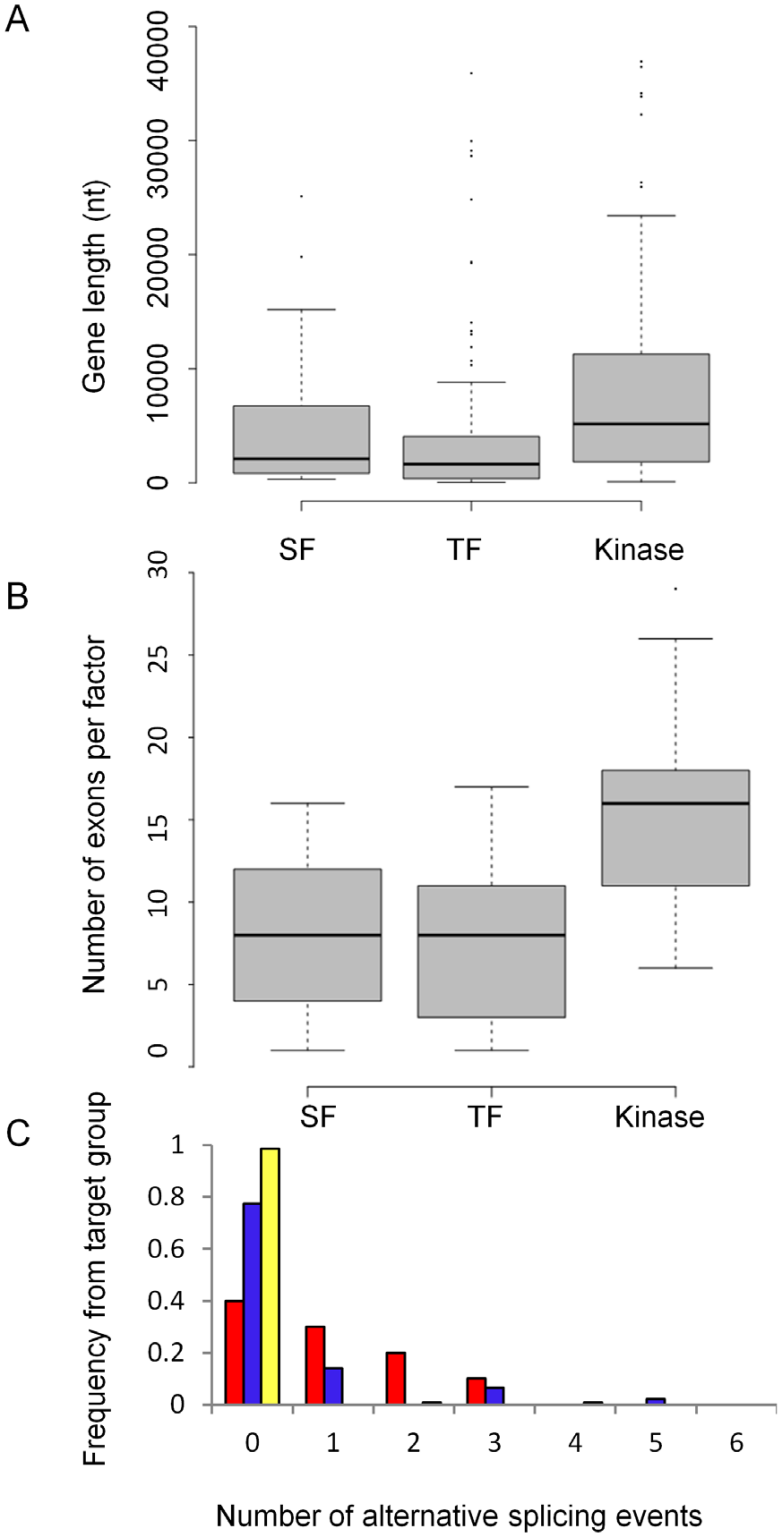
Distribution of gene length, exon number and alternative splicing events among the network genes. (A) Gene length, (B) number of exons per gene and (C) the histogram representing the normalized frequency of AS events per gene based on [70] for the three subgroups of network targets: SFs (red), TFs (blue) and kinases (yellow).

It was previously suggested that TF genes undergo, on average, more AS events compared to other human genes. In a recent comparative genomic study of the human and mouse genome, it was shown that approximately 30% of human TFs and 20% of mouse TFs had at least one isoform with a different domain composition, the DNA-binding domains being the most variable domain. These data suggested that the specific function of TFs and their expression levels are highly determined both by AS and transcriptional regulation [9]. We compared the number of alternative spliced isoforms for the different groups of regulatory proteins in our network. As shown in Figures 2 and S1, while the median length and number of exons do not differ significantly between SFs and TFs (*p-value* = *0.2*, Mann-Whitney (MW) test), SFs had significantly more AS events per factor both when the AS events were derived from Dataset A, which was based on splicing sensitive microarrays (*p-value = 6e-4*, MW test), and from Dataset B, which was derived from RNAseq data (*p-value = 4.5e-6*, MW test). Interestingly, while the kinase genes in the network were found to be significantly longer having a larger number of exons compared to SFs and TFs (*p-value = 8.6e-8, 5.2e-11*, respectively, MW test), the number of AS events per kinase was still, on average, much lower than for SFs (Figure 2 and S1).

### Splicing versus transcriptional regulation density among the network's subgroups

**Figure 3:**
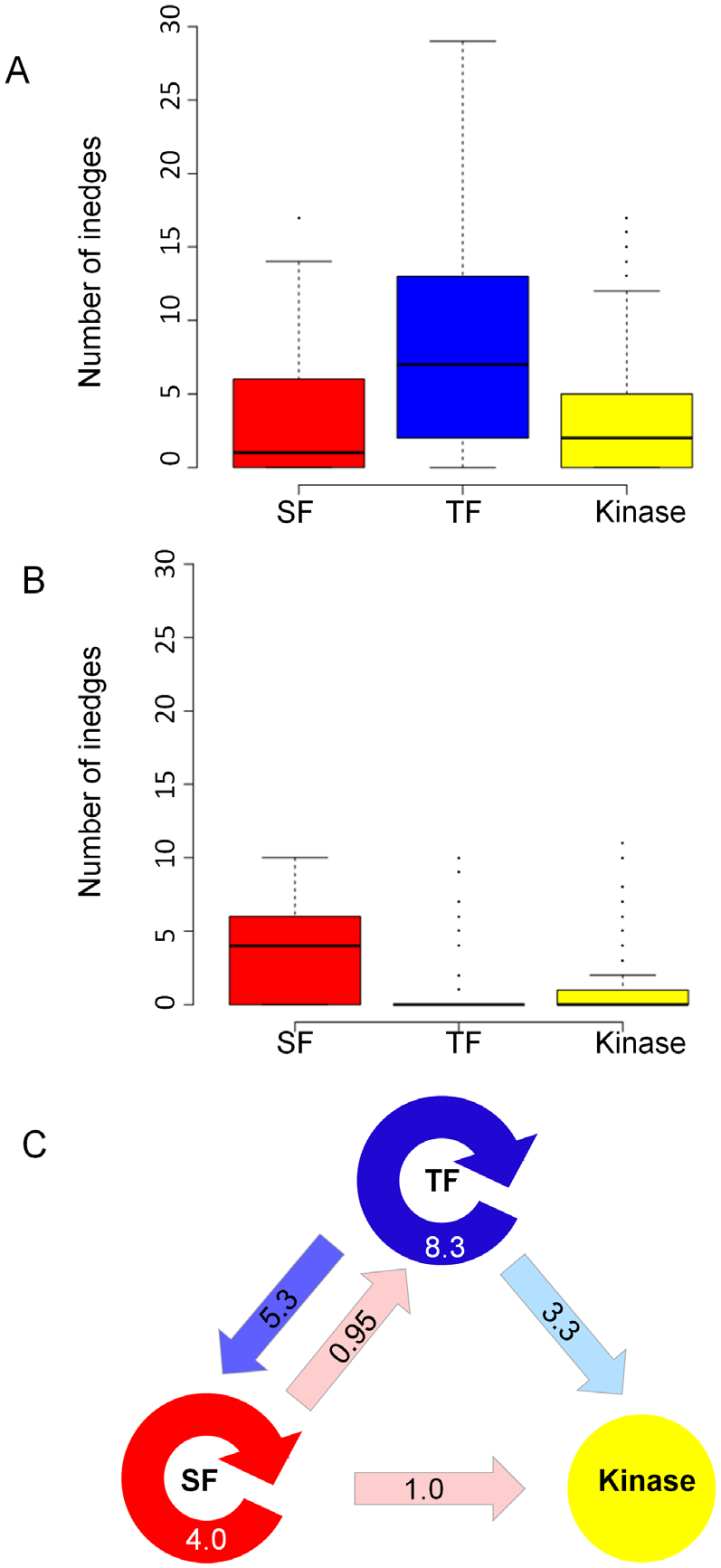
Density of transcription and splicing regulation inedges. (A) Distribution of transcription regulation inedges for the three subgroups of network targets: SFs (red), TFs (blue) and kinases (yellow) (network reconstructed based on Dataset A). (B) Distribution of splicing regulation inedges for the three subgroups: SFs (red), TFs (blue) and kinases (yellow). (C) A diagram summarizing the transcription and AS predicted interactions among the three subgroups in the network; the arrows represent interactions across and between subgroups (blue and red arrows for transcriptional and splicing regulation, respectively). The average density of inedges per group is shown in numbers within the arrow and is represented by the color intensity of the arrow. As demonstrated, cross-regulation is far more prevalent than cross-talk for both transcriptional and AS regulation.

We further examined the inedge density among the different protein subgroups, first analyzing the transcription and AS edges independently. As illustrated in Figure 3A and detailed in Dataset S1, we found a significantly higher density of transcription inedges per TFs compared to their density towards the other subgroups (*p-value = 1.2e-3 and 3.8e-7* when comparing to SFs and kinases, respectively, MW test). Interestingly, in a previous study by Balaji et al. [17] in which a combinatorial network of TFs was analyzed in yeast, the authors noticed a similar trend of co-regulatory association of TFs to the subgroup of TF genes in their network. Strikingly, the same phenomenon was found in our integrated network for AS regulation; here, we observed a significantly higher density of splicing inedges towards SFs compared to other nodes in the network (Figure 3B and Dataset S1). Specifically, we noticed a significant difference between splicing inedge density to SFs relative to TFs (*p-value = 2.3e-4*, MW test) as well as between inedge density to SFs relative to kinases (*p-value = 2.7e-3*, MW test). Very similar trends were observed for the network derived from Dataset B (Figure S2 and Dataset S2). When examining the kinases as a group, we noticed that the kinases exhibited a similar density of transcription inedges as the SFs (Figure 3A) while the splicing inedge density per kinase did not differ significantly from the average density per TF (Figure 3B). As summarized in Figure 3C, the average number of transcription inedges to TFs (8.3±0.75) and splicing inedges to SFs (4.0±0.7) was the highest among each type of interactions. Nevertheless, cross-talk interactions between regulatory proteins belonging to different subgroups were also observed in the network, i.e., transcription inedges to SFs and kinases (5.3±2.2 and 3.3±0.3 for SF and kinases, respectively) and splicing inedges to TFs and kinases (0.95±0.22 and 1±0.15 for TF and kinases, respectively). As demonstrated, the density of the latter interactions was significantly lower than the density of the cross-regulation interactions. To verify that the distinct distribution of the inedge density of splicing and transcription regulation between the groups differs from what would be expected by chance, we randomly selected from the network three groups of nodes of equal size from the original SFs, TFs and kinases groups. For each random group we calculated the inedge splicing and transcription distribution. We repeated the procedure 100 times and calculated average and standard deviation values for the randomly selected groups. As clearly shown in Table S1, both the splicing and transcription inedge distributions of the randomly selected groups could not be distinguished from each other. These results strongly reinforce that the significant differences observed for the functional groups (Figure 3 and Table S1) are not expected by chance and plausibly reflect inherent differences in the regulation of these different functional groups.

**Figure 4:**
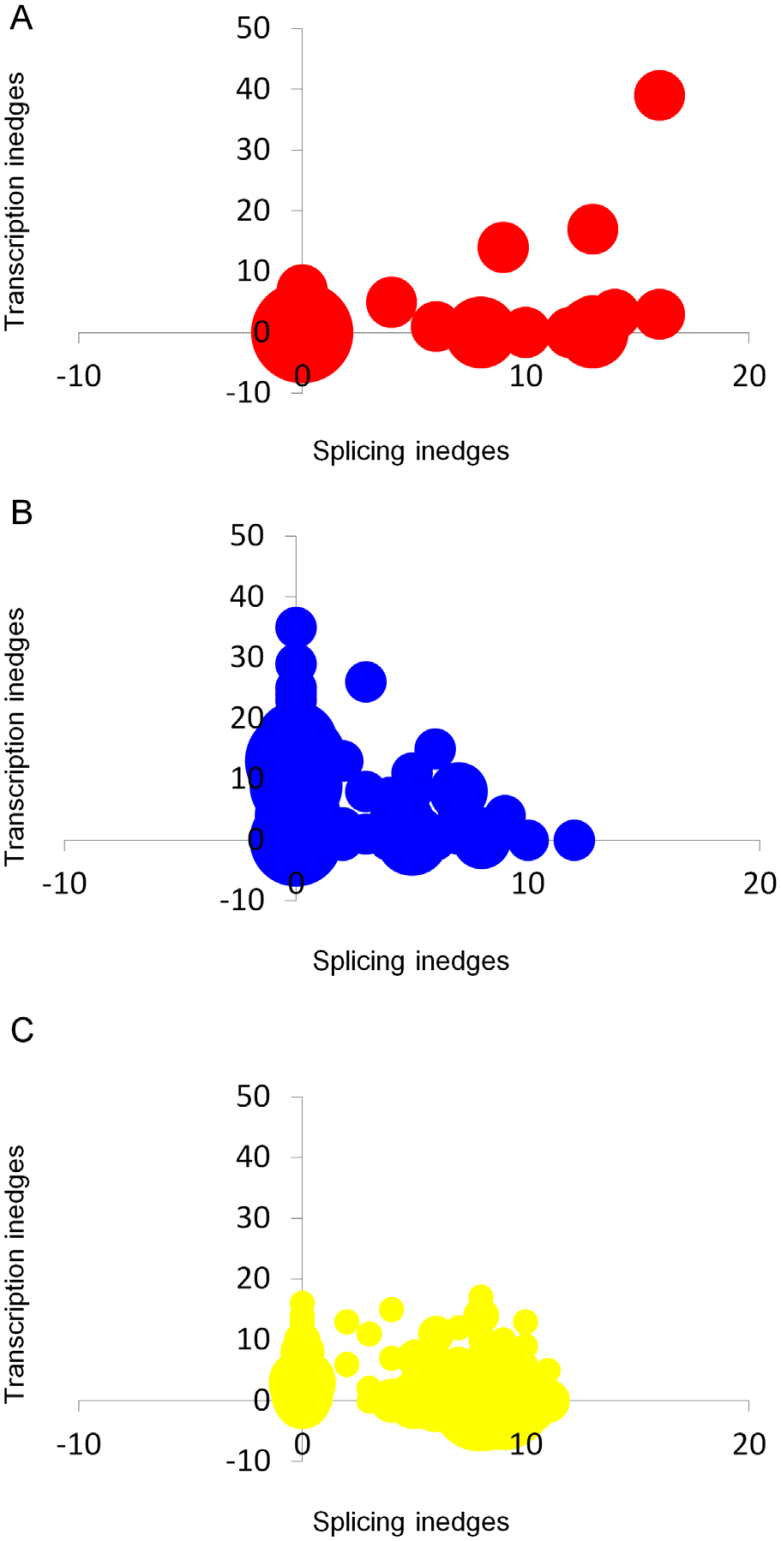
Correlation between splicing regulation inedges and transcription regulation inedges in the integrated network. Correlations are shown for the three subgroup targets: (A) SFs, (B) TFs and (C) kinases (network reconstructed based on Dataset A).

To further study the relationship between splicing and transcriptional regulation in the network, we counted the number of splicing inedges versus transcription inedges per node in each of the target groups. As illustrated in Figure 4, we noticed that the correlation between AS and transcriptional regulation differs between the different target groups. For the subgroup of SFs (Figure 4A), we observed an overall positive correlation between splicing inedges and transcription inedges towards the targets within the subgroup (*ρ* = 0.3, Spearman's rank correlation (SC)). Whereas, when considering the subgroup of TFs (Figure 4B), we noticed a weak negative correlation (*ρ* = −0.25, SC), i.e., a factor regulated by higher density of transcription inedges has a weaker density of splicing inedges, and *vice versa*. Finally, consistent with the previous analysis, we noticed an overall lower density of splicing and transcription inedges towards the subgroup of kinases (Figure 4C) with a weak negative correlation (*ρ* = −0.18, SC). Similar results were obtained for Dataset B (see Figure S3).

**Figure 5:**
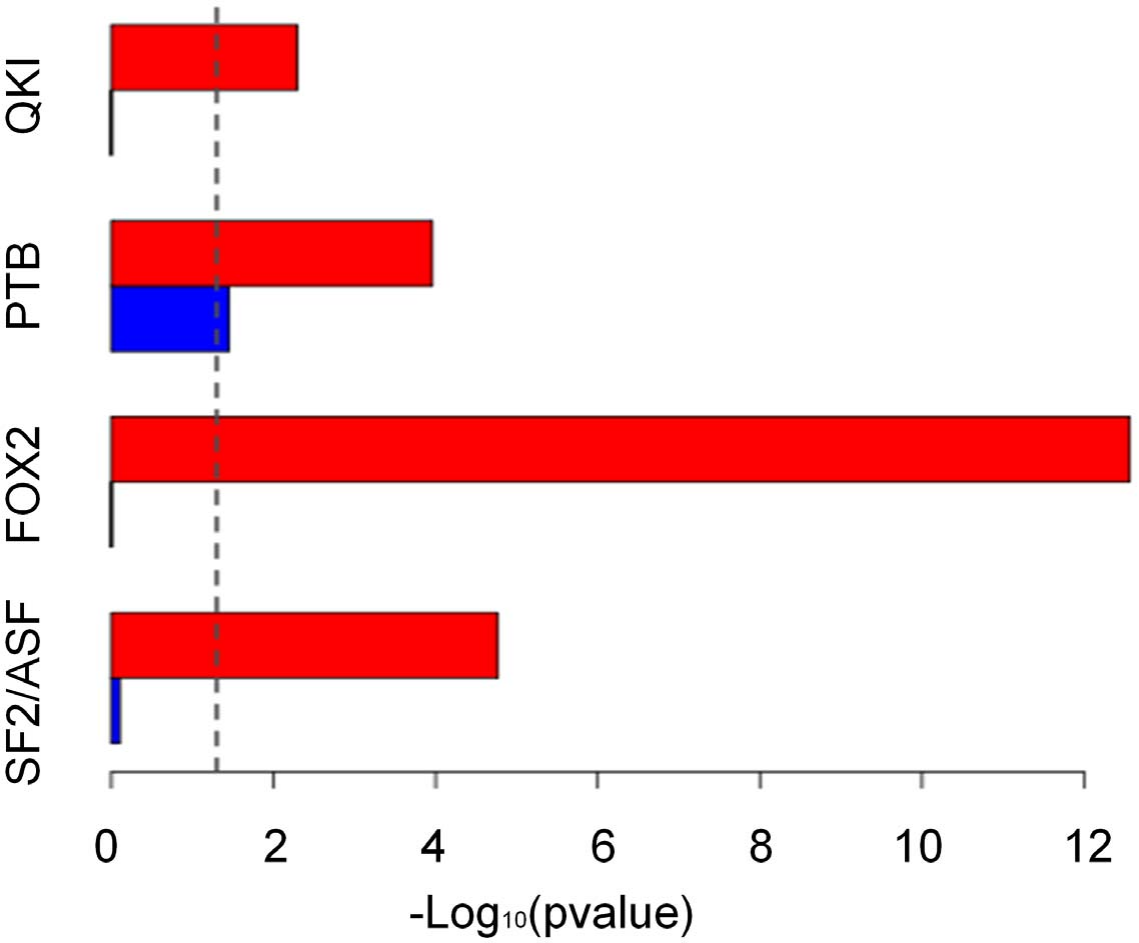
The GO term “RNA splicing” is significantly enriched among experimentally verified targets of SFs. GO enrichment for CLIP targets of four SFs, SF2/ASF (HEK 293 cells), FOX2 (Embryonic Stem Cells), PTB (HeLa cells) and QKI (HEK 293 cells) for two GO annotation terms: transcription factor activity (blue) and RNA splicing (red). Bar height represents the statistical significance shown by–log10 of the P-value. The dashed line marks the level of statistical significance (*p-value = 5e-2*).

We next asked whether the trends we noticed in the integrated network are supported by experimental binding data. To this end, we searched for enrichments of GO annotations among the targets of SFs and TFs derived from available Cross-Linking and ImmunoPrecipitation (CLIP/CLIP-seq), photoactivatable ribonucleoside-enhanced CLIP (PAR-CLIP) and chromatin immunoprecipitation-sequencing (ChIP-seq). As shown in Figure 5, we found that the GO term “RNA splicing” was enriched significantly among experimentally verified targets of SFs (*p-values* = *1.1e-4, 2.7e-13, 1.7e-5, 5e-3* for PTB [31], FOX2 [36], SF2/ASF [29] and QKI [32], respectively) while transcription activity was only weakly enriched for PTB targets (*p-value* = *3.5e-2*). To verify that the enrichment of the “RNA splicing” term among the SFs targets in the experiments is not the result of a potentially higher abundance of splicing related proteins in the data, we took as control the binding targets of the RNA-binding protein Human Pumilio 2 (PUM2) extracted from the same cells as the binding targets of QKI were extracted (Human embryonic kidney (HEK) 293 cells) [32]. In the latter case, we did not observe a statistically significant enrichment of the “RNA splicing” term (*p-value = 6e-2*), supporting that the enrichment of splicing related proteins among the SFs targets truly reflects the extensive cross-regulation among this regulatory protein family. We further analyzed ChIP-seq data from the ENCODE project [37] for nine TFs that were included in our network. Here, no significant enrichment was observed for any of the above specified GO terms.

### Splicing versus transcriptional regulation density in tissue-specific subnetworks

The integrated network described above represents putative regulatory interactions (i.e., splicing and transcription) among the three regulatory protein groups. Clearly, only one subset of the interactions is expected to take place in a given tissue or at an explicit developmental stage depending on the spatial and temporal expression of the factors. To test whether the general trend of pervasive cross-regulation observed in the network can be detected when considering only interactions between factors expressed in the same tissue, we constructed tissue-specific integrated subnetworks for two different tissues, heart and smooth muscle, in which we found the largest subset of factors expressed above the background (see Materials and Methods section and Dataset S2). Overall, the heart subnetwork included 33 TFs and 14 SFs, while the smooth muscle subnetwork included 40 TFs and 11 SFs. As shown in Table 1, consistent with the results of the large integrated network, in both tissue-specific networks we observed a higher density of splicing regulation towards the SFs while transcription regulation inedge density was higher among the TFs. Notably, due to the small sample size and the high diversity in the inedge density among the factors, statistical significance was detected only for splicing regulation within the smooth muscle subnetwork using Dataset B (*p-value = 8e-3*). Nevertheless, the general trend of cross-regulation *vs.* cross-talk regulation was clearly observed among all tissue-specific subnetworks.

**Table 1:** Inedge average density for heart and smooth muscle specific subnetworks.

Regulation type	Splicing Regulation		Transcription Regulation	
Target type	SF	TF	SF	TF
Muscle (Dataset A)	4.00±1.5	2.88±0.6	0.79±0.3	1.53±0.4
Muscle (Dataset B)	7.1±0.9	5.22±1.3	0.42±0.2	2.70±0.8
Heart (Dataset A)	6.33±0.3	2.88±0.5	1.82±1.2	2.08±0.4
Heart (Dataset B)	6.00±1.5	3.20±0.9	1.00±0.7	1.72±0.5

### Combinatorial regulation of SFs and TFs as detected from the integrated network

**Figure 6:**
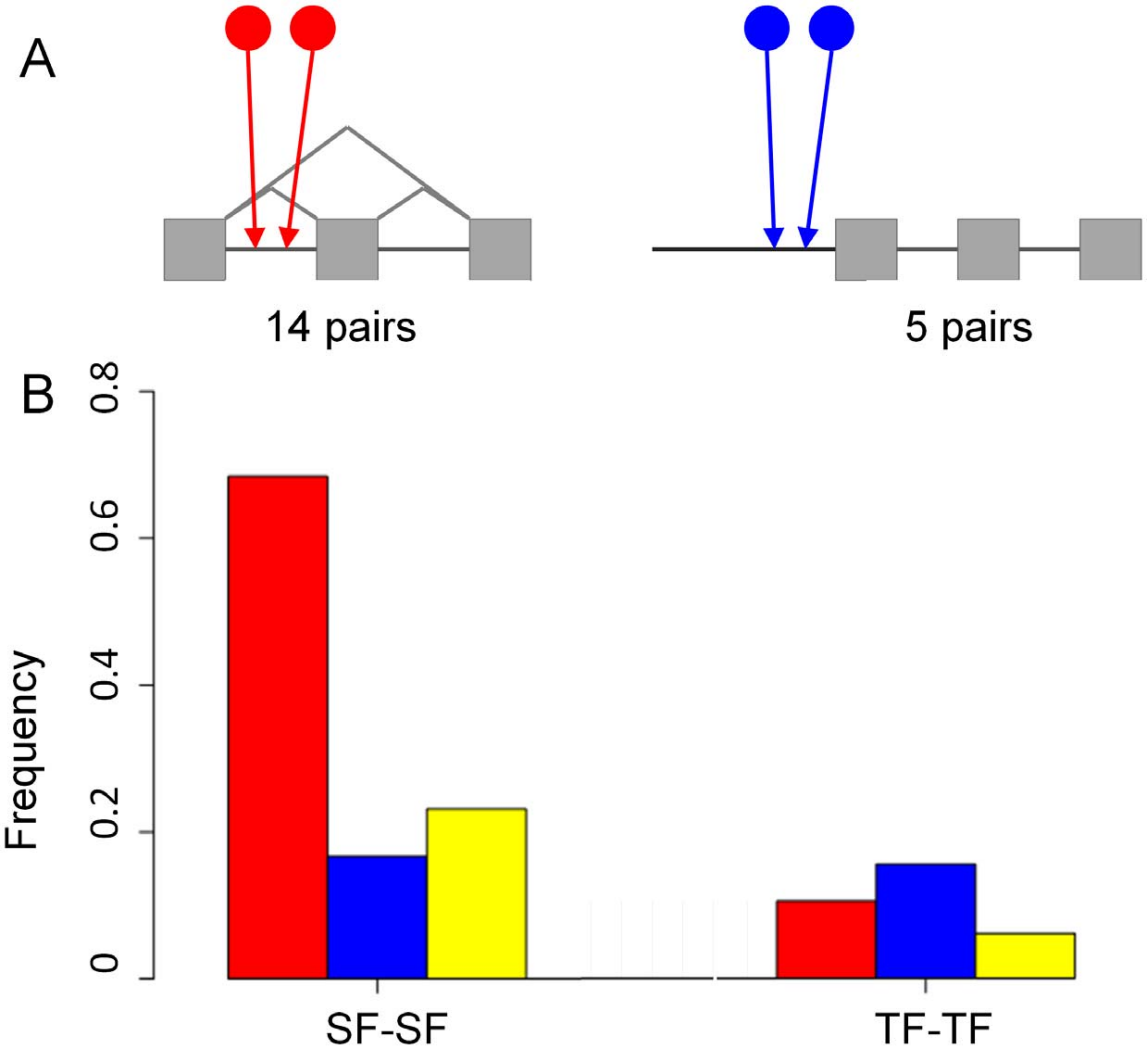
Combinatorial regulation in the integrated network. (A) Sketch describing the combinatorial relations between SFs (red, on the right) and TFs (blue, on the left), and the number of combinatorial pairs (*p-value<1e-16*) found in the network (network reconstructed based on Dataset A). (B) The frequency of genes in each subgroup target: SFs (red), TFs (blue) and kinases (yellow) regulated by the significant pairs in A: SF-SF (left) and TF-TF (right).

Previous high-throughput studies have pointed to extensive coordinated regulation both at the transcriptional and post-transcriptional levels (as reviewed in [2]). We searched for three combinatorial binding types: a combination of specific SF-SF, TF-TF and SF-TF pairs. We mapped the binding sites of all TFs and SFs for each of the factors in our network and calculated the preferences for all possible pairs to bind the same targets (see Materials and Methods section). Overall, we detected 14 different pairs of SF-SF and five pairs of TF-TF that were connected to the same genes in a coordinated manner (Figure 6). Interestingly, we did not detect any preferences of SF-TF pairs to bind in a coordinated manner, even after lowering the stringency cutoff. Very similar results were obtained when performing the analysis on the network constructed based on Dataset B, with 27 and five significant SF-SF and TF-TF pairs, respectively (see Figure S4). While in some cases we did notice a weak sequence similarity between the binding motifs of the factors that were found to regulate the same target preferentially, in the majority of cases, the binding motifs of the different factors within the pair had no overlap. Overall, the SF subgroup had the highest fraction of genes (70%) connected by SF-SF pairs, while TF gene subgroup had the highest fraction of genes (16%) regulated by TF-TF. In the case of kinases, approximately 30% of the group was targeted by TF-TF (23%) and SF-SF pairs (6%). Taken together, 80% of the SF subgroup was connected in a coordinated manner by the significant pairs (SF-SF and TF-TF). As demonstrated in Figure 5, the fraction of all genes suggested to be regulated in a coordinated manner by TF-TF pairs was much lower than in the case of AS regulation by SF-SF pairs. Among the preferred pairs regulating the SF group, we found several genes that were documented previously to regulate splicing in a coordinated manner. For example, Htra2β and YB-1 were found to act together in regulating the inclusion of exons v4 and v5 of *CD44* [38]. Another example is the TF-TF pair BRN2 and OCT1, which was found to co-regulate TF targets preferentially; this pair was previously shown to regulate the transcription of the human *GnRH* gene [39].

### Phosphorylation site predictions among the different target subgroups

**Figure 7:**
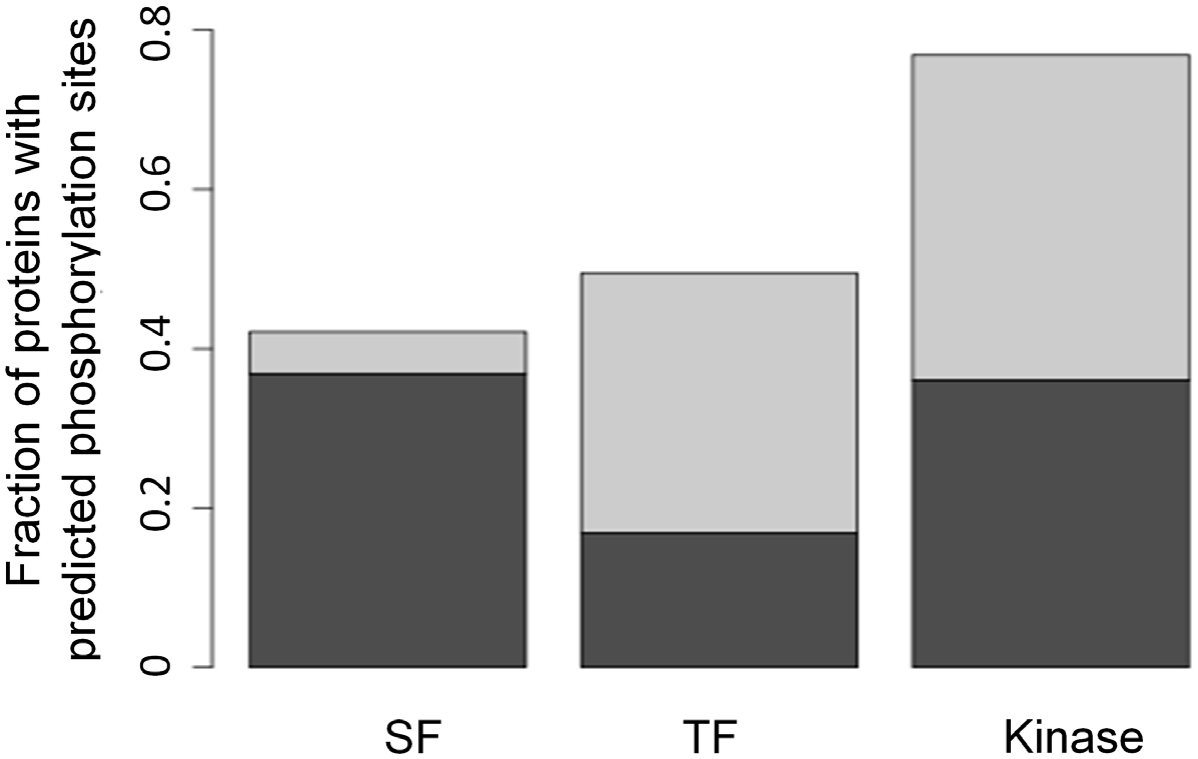
Predicted phosphorylation sites for proteins belonging to the three subgroups SFs, TFs and kinases. Height of the bars represents the frequency of proteins from each target group predicted to possess at least one phosphorylation site according to DisPhos using the “exact fragment” stringency level. The subset of proteins in each group for which the coverage of the predicted phosphorylation sites was over 10% of protein length is highlighted in dark gray.

Overall, an analysis of our integrated network revealed an interesting regulatory relationship between AS and transcription, with a clear tendency of SFs to be more densely regulated by AS whereas TFs were controlled more by transcriptional regulation. An interesting conjecture is that regulatory proteins in general tend to be regulated by the specific regulation they conduct. We were thus intrigued to examine whether this is also true for the third regulatory protein group in the network, namely the kinases. As mentioned above, due to a lack of accurate predictive methods to uniquely connect a specific kinase to its target, phosphorylation regulation could not be added as another layer of regulation to the network. Nevertheless, we could evaluate the phosphorylation regulation of the different subgroups in the network by predicting the density (normalized to the protein length) of phosphorylation sites along the protein sequences belonging to the different subgroups. Consistent with the previous findings, we found 77% of the kinases had at least one predicted phosphorylation site compared to 49% and 42% for SFs and TFs, respectively. As shown in Figure 7, while only half of the proteins in the SF were predicted to possess at least one phosphorylation site, in the majority of these proteins (88%), the region of predicted phosphorylation sites covered more than 10% of the entire protein length. As expected, the predicted phosphorylation sites in the latter group were mainly in the SR domain, which is well documented to be highly regulated by phosphorylation. Nevertheless, as a group, the kinases had the highest density of predicted phosphorylation sites suggesting tight post-translational regulation of their activity.

### Protein disorder supports extensive regulation of nodes in the network

**Figure 8:**
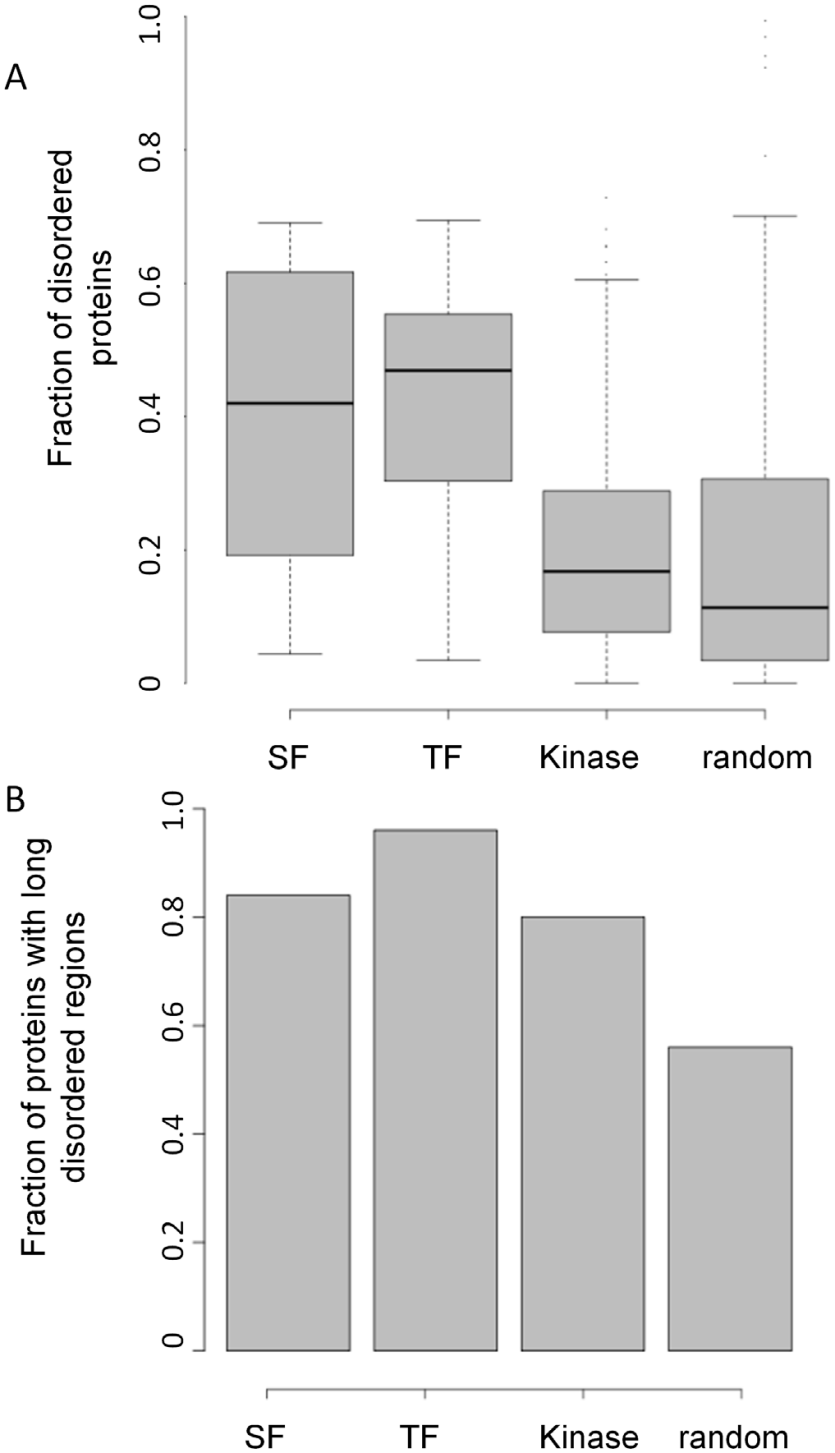
Protein disorder among the nodes of the network. (A) Frequency of predicted disordered residues per protein length for SFs, TFs, kinases and random human proteins. (B) Fraction of proteins with long disordered regions among the SFs, TFs, kinases and random protein subgroups.

It has been previously postulated that regulatory proteins would be intrinsically disordered, enabling their interaction with a large number of diverse targets (as reviewed in [40]). Indeed, it has been confirmed in human and yeast that TFs tend to be more disordered relative to other proteins in the proteome [41], [42]. In addition, the amino acid composition and sequence complexity of splicing factors from the SR protein family were found to be very similar to other disordered proteins [43]. In an earlier study, it was also shown that proteins translated from genes undergoing AS tend to be disordered, enabling structural diversity among the different protein isoforms [44]. Interestingly, kinases were found to be two-fold less disordered compared to other regulatory proteins [45]. We calculated the disorder propensity of the proteins in our networks belonging to the three regulatory groups, comparing them to random set of proteins in the human proteome (see Materials and Methods section). As demonstrated in Figure 8, and consistent with previous studies, we found that the splicing and transcription factors in our network were significantly more disordered compared to the kinases, as well as when compared to a random set of human proteins (*p-values* = *4e-4, 1e-4* for SFs versus kinases and SFs versus random set, respectively, and *2e-16* for both TFs versus kinases and TFs versus random set; MW test). Similar trends were obtained both when calculating the average number of predicted disordered residues per protein in each target group (Figure 8A) and when considering the overall fraction of disordered proteins in each subgroup (i.e., defining a protein as disordered if it included a stretch of minimal 30 disordered residues) (Figure 8B). Overall, our results confirm that the proteins in the integrated network are intrinsically disordered, specifically the TFs and SFs. This is in agreement with the high density of splicing and transcriptional regulation we observed towards the SFs and TFs subgroups in the network, which we found to be tightly controlled by their own regulation.

## Discussion

Recent high-throughput experiments and genome-scale analyses have greatly increased our understanding of the interplay between different steps of the gene expression pathway, revealing extensive coupling and coordination between transcriptional and post-transcriptional regulation [2]. Studying the cross-talk between transcriptional and splicing regulation is thus crucial for unraveling the complex gene expression regulation in higher eukaryotic organisms. The most apparent observation from the human integrated regulatory network we reconstructed in this study is the noticeable preference of regulatory proteins to be regulated via the specific regulation they conduct, namely cross-regulation. Specifically, we observed that transcription inedges were significantly denser towards the subgroup of TFs compared to the transcription inedge density towards SFs and kinases, while the splicing inedges were much denser toward the subgroup of SFs compared to TFs and kinases. These results suggest that cross-regulation among regulatory factors predominates over the regulatory interactions between the different functional groups(cross-talk).

SFs have been previously shown to autoregulate the expression of their own transcripts via splicing regulation, as well as to be cross-regulated by AS [46], [47]. The most well-known example is the autoregulation of Sxl involved in sex-fate decisions in *Drosophila* [48]. Among the splicing regulation interactions in our integrated network, we identified many experimentally verified autoregulations of SFs such as for SC35 [49], SRp20 [50], 9G8 [51], Htra2-beta [52], PTB [53] and NOVA [54]. We also identified putative interactions, which, to the best of our knowledge have not yet been reported, such as the predicted autoregulation of QKI. In addition, we detected many known interactions between different SR proteins, for example, the interactions between SF2/ASF and SRp20 that have been shown to antagonize the autoregulation of SRp20 [50], as well as interactions between SFs belonging to different protein families, such as the validated interaction between hnRNPH/F and SC35 [55] and between QKI and SF2/ASF [56].

Based on the relatively high number of AS events in gene coding for SR proteins and the extremely high conservation of their alternative exons, it has been previously suggested that AS plays a critical role in the regulation of SR protein transcripts across multiple eukaryotic lineages [57]–[59]. While many studies have pointed to the general tendency of SFs to regulate other SFs [47], our study is the first comprehensive analysis showing the significant preference of AS regulation towards SFs compared to other regulatory proteins. The prominent mode of regulation for SFs to regulate genes involved in splicing is also supported by RNA-binding data from recent CLIP/PAR-CLIP experiments conducted in human cell lines in which we found a significant enrichment of splicing-related GO annotations among the targets of four different SFs. This is consistent with recent high-throughput RNA-binding studies that noticed overrepresentation of RNA processing factors among the targets of SFs (as, for example, SF2/ASF [60]). Furthermore, indirect evidence of the tendency of SFs to regulate splicing-related genes has been found in other species. For example, in *S. cerevisiae* it has been shown that the knockdown of SFs predominantly downregulated the expression of splicing-related genes [5].

In addition to the noticeably higher inedge splicing density of the SF subgroup compared to TFs and kinases in our integrated network, our data suggest that SFs as a group are generally more regulated, both independently and via combinatorial regulation. The high density of inedges towards SFs in the network is also supported by the greater number of exons in the genes within this subgroup and their high disorder propensity. Moreover, we observed a strong preference of pairs of SFs and TFs to be connected to other regulatory proteins in a coordinated manner. These results are again in agreement with many recent studies suggesting an important role played by coordinated binding of transcription [17], [24], [61] and splicing factors [62]–[64] on their mutual targets. Combinatorial regulation may offer elegant solutions for a quick cellular response when cell conditions change or for the integration of different signals. In addition, combinatorial binding can contribute to expanding the functional diversity achieved by AS [65]). Here, we propose that combinatorial regulation by SFs is specifically widespread among regulatory proteins. More so, our results support that the SFs themselves are significantly more controlled by combinatorial regulation in comparison to other groups of regulatory factors. We postulate that SFs tend to tightly control regulatory genes at the post-transcriptional level in a coordinated manner as a possible mechanism for their role in ‘fine-tuning’ the gene expression regulation.

Overall, consistent with many examples of feedback regulation in the gene expression pathway (such as in the sxl example [48]), our data suggest that cross-regulation among the master regulators of the pathway is highly predominant. This phenomenon was also strengthened by phosphorylation site prediction analyses we conducted on the proteins (nodes) belonging to the different subgroups in the network, demonstrating that kinases as a group are more tightly regulated by phosphorylation in comparison to transcription and splicing factors. These latter results are in agreement with the well-known knowledge that kinases self-modulate each other's function and activity through phosphorylation events [66] and are consistent with recent large-scale proteomic analyses showing significant enrichment of kinases in the human kinome [67]. The prevalent cross-regulation within the functional groups observed in our integrated network can explain recent findings showing distinctive functional characteristics (mRNA and protein half-lives) for each of the regulatory groups in the network; proteins involved in transcriptional regulation having unstable mRNA and unstable proteins, proteins regulating RNA splicing having unstable mRNA and stable proteins; and proteins involved in phosphorylation having stable mRNA and unstable proteins [68]. Our network results showing that the different members within each group tend to be regulated by the same cohort of regulators is consistent with the experimental observations that they all tend to have the same expression pattern (i.e., mRNA stability and protein levels). Taken together, the network results and the experimental observations from the transcriptomic and proteomic data support the hypothesis that these regulatory protein groups are consistently under similar regulatory constraints. Notwithstanding, in addition to the tendency for extensive cross-regulation within each subgroup, we observed a significant number of interactions between factors (i.e., SF regulating TF via alternative splicing and *vice versa*). Among these interactions, we observed a putative splicing regulation between the SF SRp55 and the TF *Pax6* known to regulate eye development in vertebrates. An interaction between the D. *melanogaster* SR protein B52/SRp55 and eyeless (the *Drosophila* homolog of Pax6) has been previously shown to control eye organogenesis and size in *Drosophila* [69]. Interestingly, based on our network, we predict that the human *Pax6* gene is also regulated by the SR protein SF2/ASF while Fic et al. could not confirm the homologous interaction in *Drosophila* [69]. Overall, we predict many putative interactions in the network between SFs and TFs, arguing that this type of cross-talk regulation may play a unique role in the gene expression pathway, for example, in directing stem cell pluripotency [10] or deriving a specific developmental program [11]. While cross-talk interactions were clearly less abundant in our network, we postulate that they may be key players in tissue specificity and development. Clearly, modeling and testing other integrated networks of regulatory factors in different human tissues and other species will be required to better understand the relative contribution of cross-regulation and cross-talk interactions to modulating gene expression in high eukaryotic systems.

## Materials and Methods

### Network construction

#### Integrated network

The SF group was comprised of 20 most extensively studied SFs belonging to the two major protein families, SR proteins and hnRNPs, for which experimental information on their binding sites was available [28]. An edge from SF_i_ to any other factor j (where i runs from 1 to 20 and j from 1 to 257) was added if factor j had an AS event and a human-mouse conserved binding motif of SF_i_ was found flanking the splicing event region. The splicing events in the network were defined based on two independent datasets: “Dataset A” based on expression data from splicing microarrays from Castle et al. [70] and “Dataset B” derived from RNA-seq data from Katz et al. [71]. The SFBSs were predicted using the SFmap [28] algorithm with medium stringency (see http://sfmap.technion.ac.il/manual.html). Three types of AS events were considered: cassette exon, alternative 3′ and alternative 5′. For cassette exon events, splicing motifs were searched within 100 nt of the immediate upstream intron, the entire exon and 100 nt of the flanking downstream intron. For alternative 3′ splicing events, splicing motifs were searched within 100 nt of the upstream intron and the entire exon flanking the event. In alternative 5′ splicing events, motifs were searched in the entire exon and 100 nt of the flanking downstream intron.

The TF nodes in the network were chosen from the human-mouse-rat conserved TFBS factors table of the UCSC genome browser, hg18 version, including 90 TFs that have experimentally verified motifs [33]. The transcription regulation edges were defined based on the existence of a conserved TF binding site in promoters of genes in the network based on the TFBS sites table. Thus, an edge from TF_i_ (where i runs from 1 to 90) exists only if the binding motif of that TF was found in the promoter region of any factor j (where j runs from 1 to 257). Promoters were defined as 5 kb upstream to the transcription start site of the genes (as defined in [25]). The kinase group was composed of 147 human kinase proteins downloaded from kinbase (http://kinase.com/kinbase) based on Manning et al. [26].

#### Tissue-specific networks

To construct the tissue-specific network, expression data from Human GNF1H Gene Atlas based on Human Genome U133A 2.0 Array [72] was incorporated. The expression data for each of the factors in the entire network were normalized by extracting the expression values of all genes in the tissues available from the array and calculating the average gene expression for the tissue. Factors were included in the tissue-specific network if their expression was above average. The heart and smooth muscle tissues were chosen as these tissues had the largest subset of factors (from the entire set of factors in the network) that were expressed above the average. For the heart tissue, 33 TFs and 14 SFs were included (nine and four of which had AS events, respectively). For the smooth muscle tissue, 40 TFs and 11 SFs were included (eight and three of which had AS events, respectively). Putative interactions between nodes in the network were calculated as in the integrated network.

### Calculating network properties

#### Clustering coefficient

The clustering coefficient, or transitivity, is the measurement of interactions within cliques in the network. It is a function of the number of neighbors of each node and triplets of nodes. The global clustering coefficient is the sum of all clustering coefficients C_i_, where i runs from 1 to N (the number of nodes in the network), and is defined as the fraction of closed triplets that exist among its nearest neighbors relative to the maximum number of triplet types among all neighbors. See Formula 1.
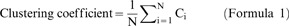
The clustering coefficient was calculated with igraph package version 0.5.4 using GNU R statistical software (http://cneurocvs.rmki.kfki.hu/igraph).

#### Sparseness

Sparseness was defined as the number of network edges (E) over the maximum number of possible edges, which is defined as the numbers of edges squared (E_max_ = E^2^). See Formula 2.
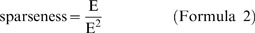
In order to test the network properties, 1,000 random networks were constructed with the same number of nodes and the same average number of edges (degree) using igraph package version 0.5.4 using GNU R statistical software (http://cneurocvs.rmki.kfki.hu/igraph).

### Gene ontology and motif enrichment analysis

#### CLIP data analysis

SF2/ASF CLIP data were taken from the supplementary data of [29]. FOX2 CLIP data were downloaded from FOX2ClipSeq table at the human genome browser [33]. PTB CLIP data were taken from [31]. QKI and PUM2 PAR-CLIP data were taken from the supplementary data of [32]. For each set of targets, we searched for GO term enrichment using DAVID [73].

To validate SFmap motifs against CLIP/PAR-CLIP data, we ran SFmap on the set of experimentally binding sequences downloaded from [31], [32] using the same parameters used to generate the network. As control we used 1,000 sequences from the middle of the introns extracted randomly from the human genome version hg18. All analyzed sequences (selected from the experiments and the control) were 41 nucleotides in length. The Fisher Exact Test (based on hypergeometric distribution) was applied to examine whether the motifs predicted by SFmap were significantly enriched in the experimentally binding sequences relative to the random set of sequences.

#### ChIP-seq data analysis

Data for the ChIP-seq analysis were taken from the wgEncodeRegTfbsClustered table of the UCSC genome browser [33] based on the ENCODE project [37]. We analyzed the data for nine TFs included in the network: USF1, NFBK, C-Myc, HNF4a, IRF4, p300, PAX5, POU2F2 and TCF12. GOrilla was further applied to search for enrichment of GO terms [74].

### Phosphorylation site prediction

Prediction of phosphorylation sites in SFs, TFs and kinases was carried out by DisPhos [75] using the “exact fragment” stringency level. The “exact fragment” stringency level is based on matching the exact fragment of 25 amino acids in another protein with a known phosphorylation site to the predicted phosphorylation site. For each protein group, the average number of proteins with at least one phosphorylation site was calculated. Furthermore, the frequency of amino acids predicted to be involved in a phosphorylation site were calculated for each protein. The number of predicted sites was normalized to the protein length.

### Disorder prediction

Prediction of disordered residues in SFs, TFs, kinases and a random set was carried out with VSL2B [76] software using 0.75 as the cutoff for disordered residue. For each protein, the average number of disordered residues per protein length was calculated. Disordered proteins were defined if they included at least one disordered continuous segment of 30 amino acids. Calculations for the random set were carried out 10 times on 250 proteins chosen randomly from uniprot http://www.uniprot.org/.

### Combinatorial regulation of SFs and TFs

The hyper geometric distribution test was used to detect preferences of pairs to co-regulate the same target genes in the network. For each pair of factors in the network (SFs and TFs), the number of targets regulated independently and by both factors was calculated. Specific pairs of factors that were found to bind the same targets preferentially were selected (*p-value* cutoff for the hyper geometric distribution test was defined as 1e-16). To compare results between the different target groups, we calculated the relative frequency of genes within each group that were found to be wired by each significant pair in a coordinated manner.

## Supporting Information

Dataset S1 Splicing and transcription inedges for each node in the integrated regulatory network.
(XLS)

Dataset S2 Normalized expression data for network genes in smooth muscle and heart tissues from the GNF atlas.
(XLS)

Figure S1 Histogram representing the normalized frequency of AS events per gene based on RNAseq data from Dataset B in three target groups: SFs (red), TFs (blue) and kinases (yellow).
(TIF)

Figure S2 Distribution of splicing regulation inedges in the three subgroups of network targets: SF (red), TF (blue) and kinases (yellow) (network reconstructed based on Dataset B).
(TIF)

Figure S3 Correlation between splicing regulation inedges and transcription regulation inedges in the integrated network. Correlations are shown for the three subgroups of network targets: (A) SFs, (B) TFs and (C) kinases (network reconstructed based on Dataset B).
(TIF)

Figure S4 (A) Sketch describing the combinatorial relations between SFs (red, on the right) and TFs (blue, on the left), and the number of combinatorial pairs (*p value<1e-16*) found in the network (network reconstructed based on Dataset B). (B) The frequency of genes in each subgroup target: SFs (red), TFs (blue) and kinases (yellow) regulated by the significant pairs in A: SF-SF (left) and TF-TF (right).
(TIF)

Table S1 Average splicing and transcription towards SFs, TFs and kinases in the integrated regulatory vs. average splicing and transcription towards SFs, TFs and kinases in randomly selected groups and their standard deviation.
(PDF)
