# Supplementary figures and images for: An Integrated Regulatory Network Reveals Pervasive Cross-Regulation among Transcription and Splicing Factors
Source: PLoS Comput Biol. 2012 Jul 26;8(7):e1002603. doi: 10.1371/journal.pcbi.1002603 (PMC3405991; doi:10.1371/journal.pcbi.1002603)

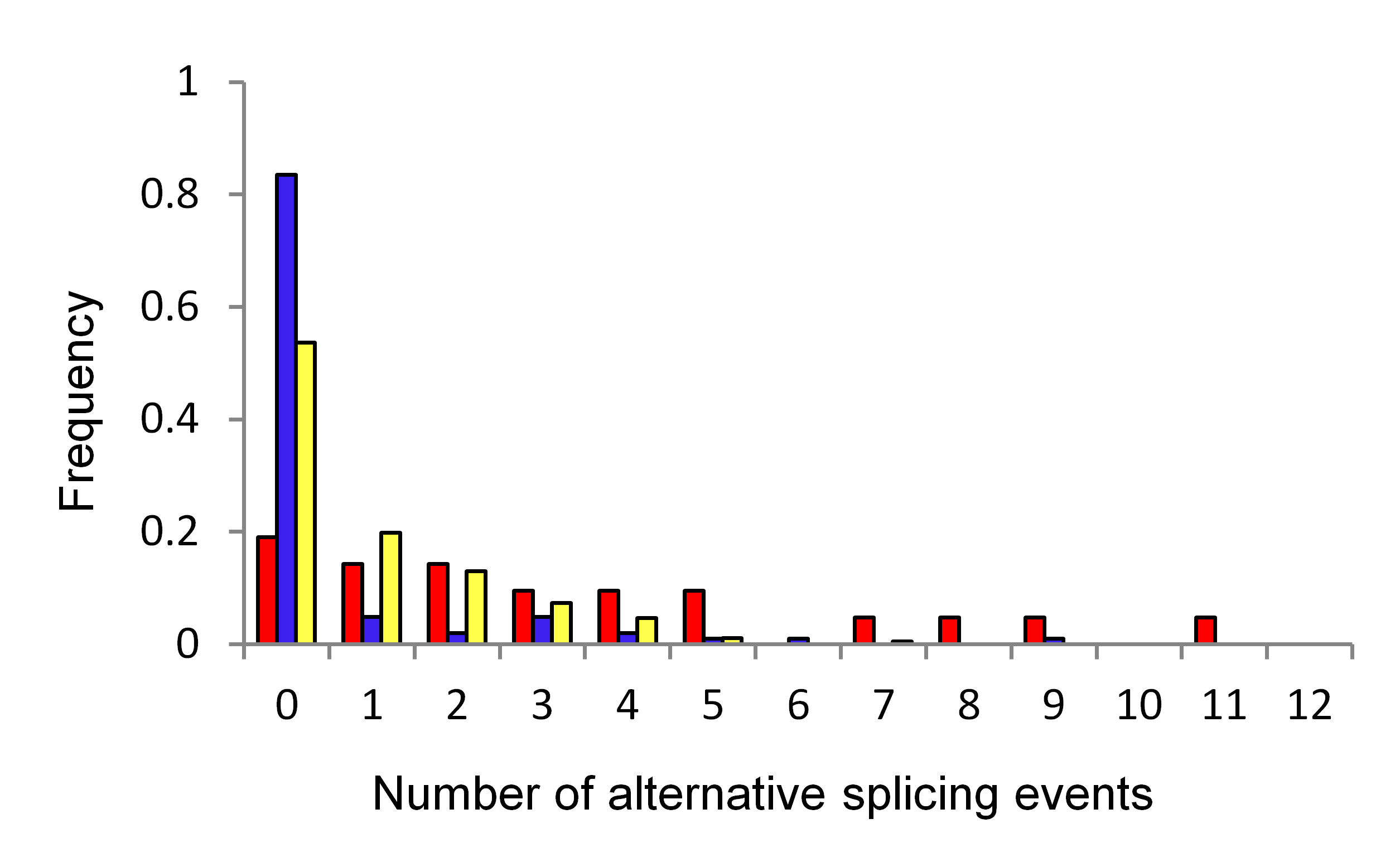

Supplement: Figure S1 — Histogram representing the normalized frequency of AS events per gene based on RNAseq data from Dataset B in three target groups: SFs (red), TFs (blue) and kinases (yellow). (TIF) [file pcbi.1002603.s003.tif]

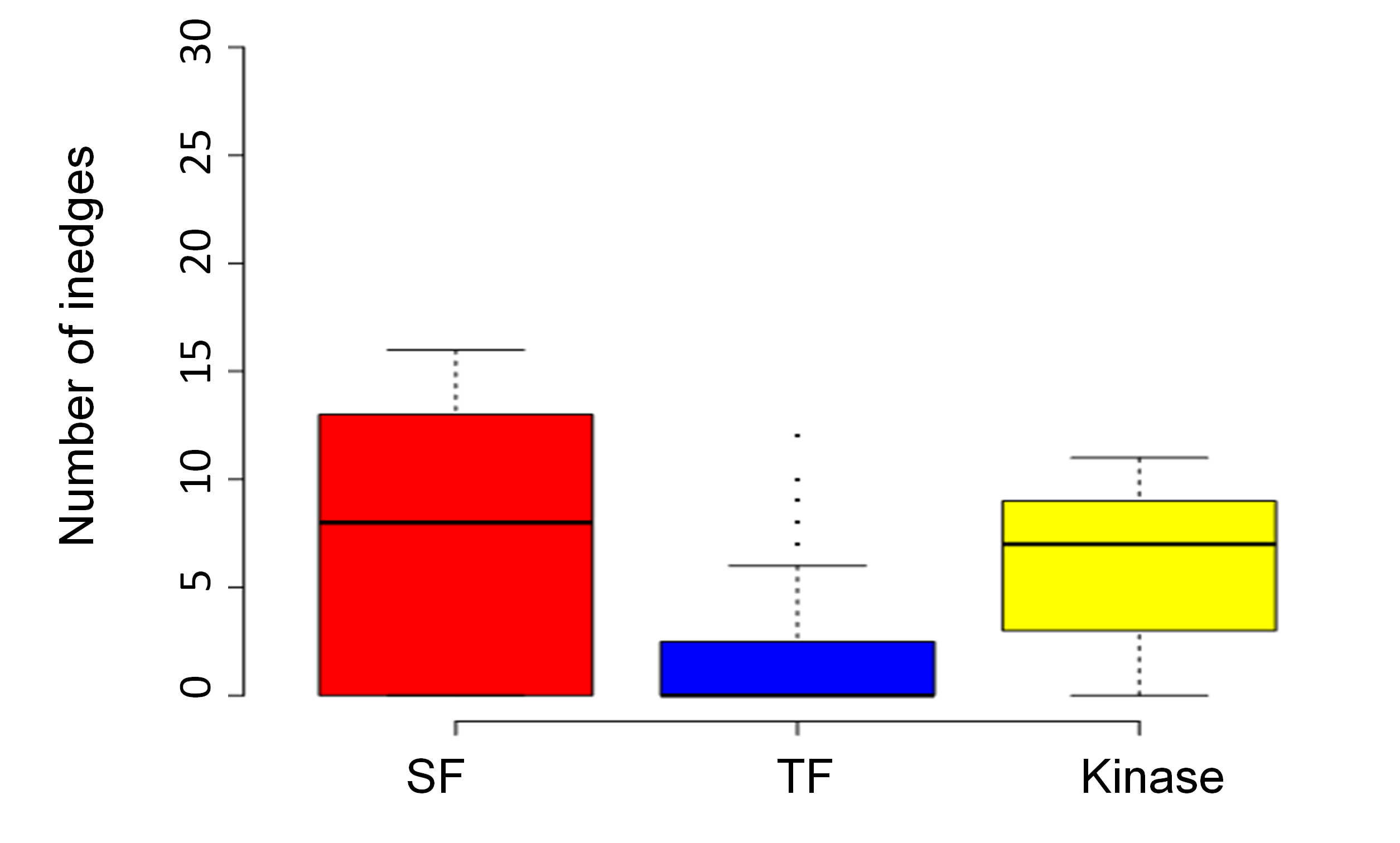

Supplement: Figure S2 — Distribution of splicing regulation inedges in the three subgroups of network targets: SF (red), TF (blue) and kinases (yellow) (network reconstructed based on Dataset B). (TIF) [file pcbi.1002603.s004.tif]

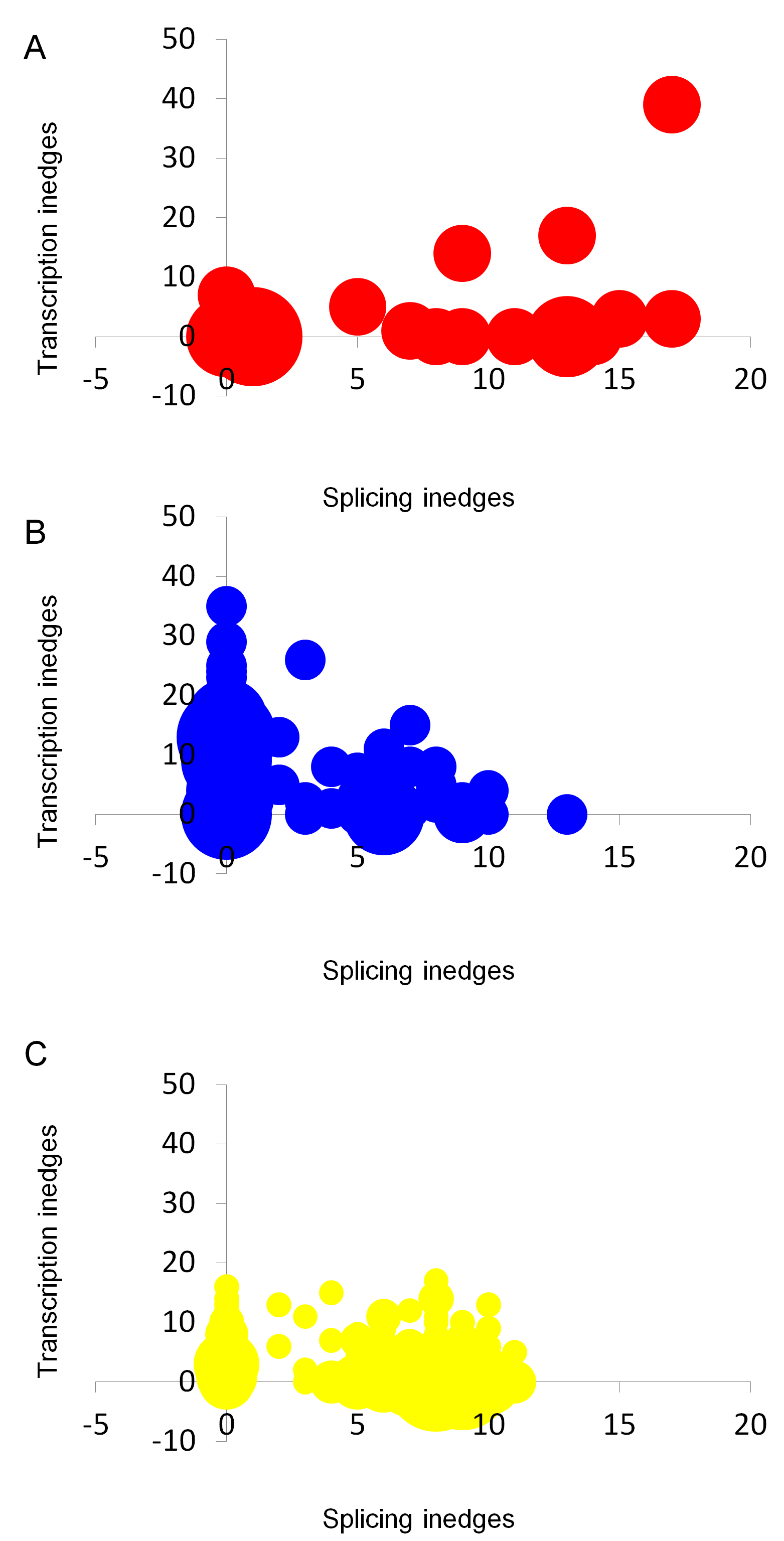

Supplement: Figure S3 — Correlation between splicing regulation inedges and transcription regulation inedges in the integrated network. Correlations are shown for the three subgroups of network targets: (A) SFs, (B) TFs and (C) kinases (network reconstructed based on Dataset B). (TIF) [file pcbi.1002603.s005.tif]

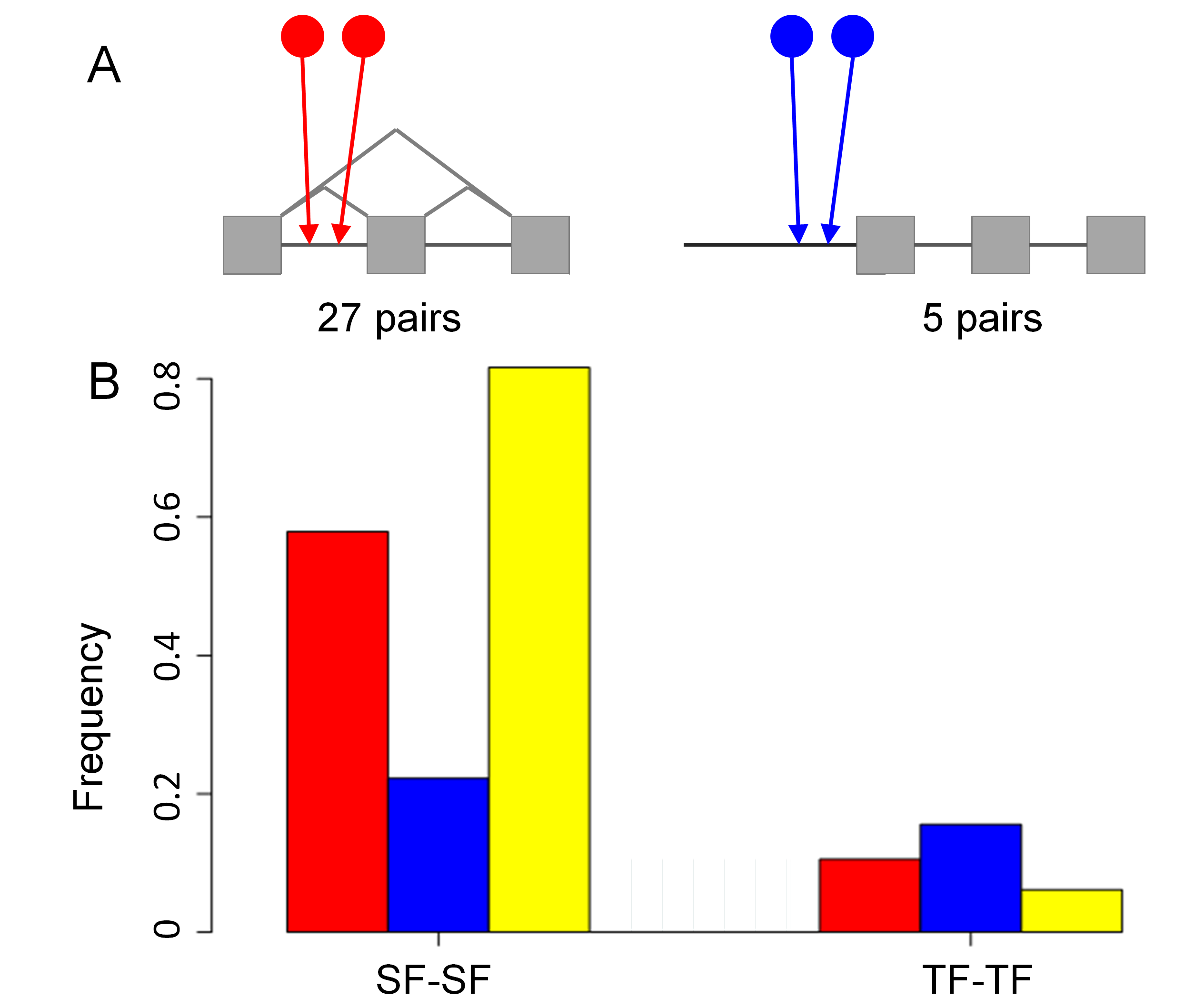

Supplement: Figure S4 — (A) Sketch describing the combinatorial relations between SFs (red, on the right) and TFs (blue, on the left), and the number of combinatorial pairs (p value<1e-16) found in the network (network reconstructed based on Dataset B). (B) The frequency of genes in each subgroup target: SFs (red), TFs (blue) and kinases (yellow) regulated by the significant pairs in A: SF-SF (left) and TF-TF (right). (TIF) [file pcbi.1002603.s006.tif]
