# Supplementary material for: An Integrated Regulatory Network Reveals Pervasive Cross-Regulation among Transcription and Splicing Factors
Source: PLoS Comput Biol. 2012 Jul 26;8(7):e1002603. doi: 10.1371/journal.pcbi.1002603 (PMC3405991; doi:10.1371/journal.pcbi.1002603)
Supplement: Table S1 — Average splicing and transcription towards SFs, TFs and kinases in the integrated regulatory vs. average splicing and transcription towards SFs, TFs and kinases in randomly selected groups and their standard deviation. (PDF) [file pcbi.1002603.s007.pdf]

Table S1 : Comparing the inedge density in the integrated network versus random networks

		Network	Random	Stdev (random)
Splicing regulation inedges	splicing factors	4	0.9	0.6
	transcription factors	0.95	1.2	0.2
	kinases	1	1.1	0.1
Transcription regulation inedges	splicing factors	5.3	5.2	1.2
	transcription factors	8.3	5.1	0.5
	kinases	3.3	5.1	0.2
